# Thresholds of ultrasound synovial abnormalities for knee osteoarthritis – a cross sectional study in the general population

**DOI:** 10.1016/j.joca.2018.09.018

**Published:** 2019-03

**Authors:** A. Sarmanova, M. Hall, G.S. Fernandes, A.M. Valdes, D.A. Walsh, M. Doherty, W. Zhang

**Affiliations:** † Division of Rheumatology, Orthopaedics and Dermatology, School of Medicine, University of Nottingham, UK; ‡ Arthritis Research UK Pain Centre, and NIHR Nottingham BRC, Nottingham, UK; § Arthritis Research UK Centre for Sports, Exercise and Osteoarthritis, Nottingham, UK; ‖ School of Health Sciences, University of Nottingham, UK

**Keywords:** Ultrasound, Ultrasonography, Knee pain, Osteoarthritis, Synovitis

## Abstract

**Objective:**

To establish “normal” ranges for synovial thickness and effusion detected by ultrasound (US) and to determine cut-offs associated with knee pain (KP) and radiographic knee osteoarthritis (RKOA) in the community.

**Methods:**

147 women and 152 men ≥40 years old were randomly selected from the Nottingham KP and Related Health in the Community (KPIC) cohort (*n* = 9506). The “normal” range was established using the percentile method in 163 participants who had no KP and no RKOA. Optimal (maximum sensitivity and specificity) and high specificity (90%) cut-offs were established using receiver operating characteristic (ROC) curve analysis in a comparison between people with both KP and RKOA and normal controls.

**Results:**

Effusion and synovial hypertrophy differed by gender but not by age or laterality, therefore gender-specific reference limits were estimated. However, the “normal” ranges between men and women were similar for effusion (0–10.3 mm vs 0–9.8 mm), but different for synovial hypertrophy (0–6.8 mm vs 0–5.4 mm). Power Doppler Signal (PDS) in the healthy controls was uncommon (1.2% in men and 0.0% in women). The optimal cut-off was 7.4 mm for men and 5.3 mm for women for effusion, and 3.7 and 1.6 for hypertrophy respectively. The high specificity cut-off was 8.9 for men and 7.8 for women for effusion, and 5.8 and 4.2 for hypertrophy respectively.

**Conclusions:**

US effusion and synovial hypertrophy but not PDS are common, but differ by gender, in community-derived people without painful knee OA. Currently used cut-offs for abnormality need reappraisal.

## Introduction

Knee osteoarthritis (OA) is a major cause of chronic pain and impaired function in older adults^1^, ^2^. Knee OA is a common complex joint disorder that involves all joint tissues including hyaline articular cartilage, fibrocartilaginous menisci, synovium, bone, ligaments and muscle^3^, ^4^, ^5^. These pathological changes can be detected using various imaging techniques such as radiographs, ultrasound (US) and magnetic resonance imaging (MRI). Because people with knee OA show wide variability of presentation with respect to compartmental involvement and degree of bony changes and inflammation, it could prove possible to use modern imaging techniques to identify potential subgroups/phenotypes in the heterogeneous population of people with knee OA^6^.

US is a non-invasive imaging technique that is used commonly to detect inflammatory changes in joints. It is relatively inexpensive, widely available and has no radiation burden or contraindications^7^. Over the last two decades a number of technical advances have improved US imaging of joints and soft tissues, increasing its utility for assessment of musculoskeletal conditions^8^. US detection of synovial effusion and synovial hypertrophy in knees is more sensitive than clinical examination^9^, ^10^, correlates well with histological findings^11^, ^12^ and correlates well with MRI in visualising effusion^13^, ^14^. However, evidence regarding “normal” values for effusion and hypertrophy in the general population is limited^15^. For example, the only study to provide reference values for effusion was based on a group of healthy volunteers aged 20–60 years old (*n* = 102)^16^, which is a low age range for OA, and no population studies have reported normal values for synovial hypertrophy or prevalence of Power Doppler signal (PDS).

A few studies have attempted to identify an optimal threshold (maximum sensitivity and maximum specificity) or scoring system for US synovial changes (USSCs) in knee OA. For example, a EULAR-ESCISIT multi-centre study involving 600 individuals with knee OA tested different cut-offs of synovial hypertrophy (≥2 mm or ≥4 mm) and effusion (≥4 mm) against radiographic severity and knee effusion on clinical examination^17^. The diagnostic accuracy of these cut-offs was low, and it was recommended that a threshold of 4 mm be used for both features^18^. Two European Multicentre Studies also found that thresholds varied depending upon knee positioning. While Terslev *et al.*^19^ found the optimal cut-off (maximum sensitivity and maximum specificity) for knee effusion detected in the neutral position with quadriceps contraction was 3.2 mm, Mandl *et al.*^20^ showed that the optimal cut-off for effusion at 30 degrees of flexion was 3.6 mm. However, the comparison in these studies was made between normal and abnormal knees in people with knee OA or other rheumatic conditions, hence the thresholds between knee OA and the general population remain unknown. Furthermore, none of the existing recommendations for scoring USSC have considered age, gender or laterality. Interestingly, the EULAR-ESCISIT study in people with knee OA noted that women had fewer joint effusions than men [OR 0.62, no confidence interval (CI) reported]^18^ but still recommended the same threshold (4 mm) for men and women. Differences in joint anatomy, physiology, pain perception and risk of incidence and progression of OA between genders provides a clear physiological basis for examining whether there is a difference in USSCs between men and women^21^.

We therefore undertook this study in a random sample of community-derived men and women aged over 40 years who are participating in a prospective cohort^22^ to: [1] examine the normal ranges of USSCs and their distributions by age, gender and laterality in the healthy participants; and [2] establish optimal cut-offs for symptomatic knee OA compared with healthy controls.

## Methods

**Fig. 1:**
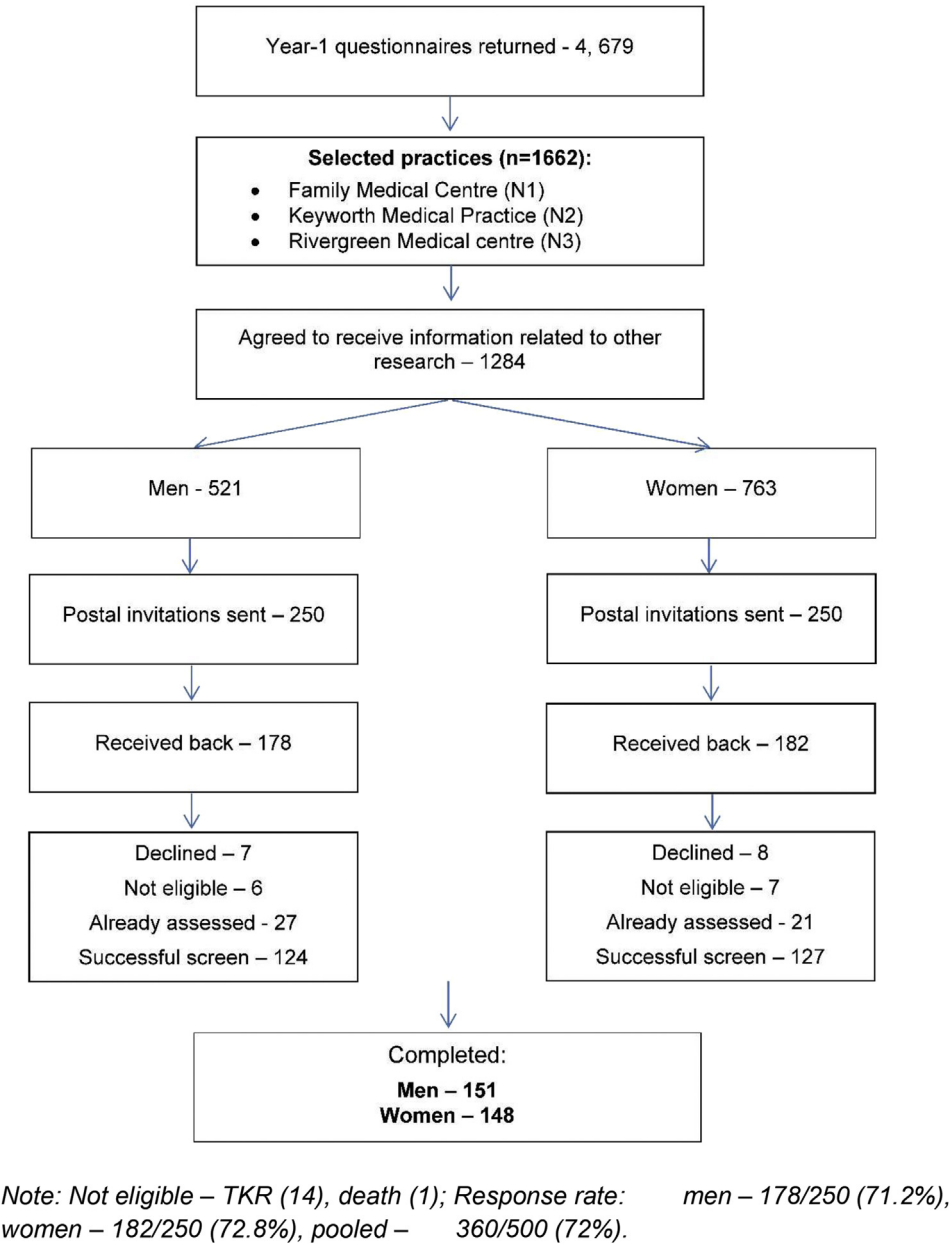
Recruitment of participants in the “Knee synovial changes detected by US in the general population: cross sectional study” (by the 28th of November 2016).

Participants for this cross sectional study were selected from the Knee Pain and Related Health in the Community Study (KPIC)^22^, an ongoing prospective cohort study in Nottingham, UK that included at baseline 9506 men and women aged ≥40 years. For convenience, participants for the KPIC were selected from the primary care practices closest to Nottingham City Hospital. To ensure that this set is representative of the whole population we compared the five selected practices with the seven unselected practices and the whole population and found no difference in terms of age, gender and body mass index (BMI) (Appendix 1). In selected practices 1662 participants replied to the follow-up questionnaire, of them 1284 (763 women, 521 men) agreed to receive information about further projects. They were stratified by gender and a random sample was taken from each group regardless of their KP/OA status (Fig. 1). The characteristics of participants invited for the current study (*n* = 500), and those who did reply (*n* = 360) are shown in Appendix 2, and characteristics of the final sample (*n* = 299) are shown in Appendix 3.

Age, gender, height, weight and KP status were self-reported in the postal questionnaire. *Current KP* was defined as pain on most days of the past month^23^, ^24^ (later referred as KP).

Participants were invited to attend for clinical assessments, including US and radiographs of both knees at Nottingham City Hospital.

1. US assessment

US examination was performed by two assessors (MH, AS), using the Toshiba Aplio SSA-770A machine with a multi-frequency (7–12 MHz) linear array transducer. The same equipment and software were used throughout the study. The supra-patellar recess and medial and lateral tibio-femoral spaces were assessed with knee flexion of approximately 20–30°. USSCs were defined according to OMERACT-7 definitions (Appendix 4)^25^. Depth of synovial thickness (hypertrophy) and effusion were each measured on a continuous scale at their maximal diameter in millimetres using the longitudinal axis. PD assessment was focused on areas of synovial hypertrophy and recorded as absent or present. All measurements were made in real time. Only one value per joint was recorded for each US feature (the maximum value across the three areas scanned). The inter-observer and intra-observer reliability test results were reported previously^26^.

2. Radiographic knee OA (RKOA) assessment

Bilateral weight-bearing semi-flexed posterior–anterior tibio-femoral views using a Rosenberg template and 30° flexion skyline patello-femoral views were undertaken using standardised protocols^22^. The Nottingham logically derived line drawing atlas (LDLDA)^27^, ^28^ was used to score joint space narrowing (JSN) in medial and lateral tibio-femoral and medial and lateral patello-femoral articulations (each scored −1 to 5) and osteophytes (at eight sites in the three compartments, each scored 0–5). The scores for all three compartments, ignoring −1 values for JSN (i.e., joint space widening), were summated as a global score for each knee. *Presence of RKOA* was defined as definite JSN (grade ≥2) plus definite osteophyte (grade ≥2) in any compartment (tibiofemoral or patellofemoral). This definition of definite osteophyte and definite narrowing accords with the pathological definition of OA which requires both definite focal loss of hyaline cartilage and definite associated bone change^29^. *Symptomatic RKOA* was defined as current KP plus RKOA. *Alternative definition* of symptomatic RKOA was as current KP plus Kellgren and Lawrence (K&L) grade ≥2 in any compartment^30^.

### Statistical analysis

1. Sample size

Sample size was calculated using the formula for a single cross-sectional study^31^. A population-based study conducted by Abraham *et al.*^32^ reported prevalence of US-detected effusion at 24%. Therefore, sample size required for this cross sectional study is 280 assuming the error margin *d* = 3%. This number also corresponds with the recommended minimum sample size for establishing reference intervals (*n* = 120 per group)^33^.

2. “Normal” range

The “normal” range was established in the healthy participants who had no KP and no RKOA^34^. We used 0 as the low limit and 95th percentile as the upper limit to define the normal range of US effusion and synovial hypertrophy. The 95% CI for the upper limit was calculated using the distribution-free method as data were not normally distributed (Hahn and Meeker, 2011).

3. Optimal threshold

The discrimination ability (i.e., ability to separate cases and controls) of each US feature was determined in a case control study, where people with symptomatic RKOA (defined above) were classified as *cases* and those with neither KP nor RKOA were classified as *controls*. Standard diagnostic accuracy measures (e.g., sensitivity and specificity, likelihood ratios (LRs)) and ROC statistics were calculated^35^, ^36^.

Two *cut-offs* were established in this study:

• An optimal cut-off with the maximum sensitivity and specificity according to the Youden index: *J* = *Maximum (Sensitivity* + *Specificity* − 1)^36^.
• A cut-off with a relatively high specificity of 90% to ensure the minimum misdiagnosis.

We also examine the sensitivity, specificity and LR etc for the 4-mm cut-off recommended by EULAR^18^. Further details regarding the statistical methods can be found in Appendix 5.

Missing data are presented in Appendix 6. All analyses were undertaken using SAS software v9.4.

## Results

### Demographic and clinical characteristics of the study population

Of the total 299, 163 individuals had no KP and no RKOA – healthy controls and 44 individuals had symptomatic RKOA. Apart from age, the healthy controls were different from symptomatic RKOA for gender, BMI, KP, radiographic score and all three US features (Table I). The prevalence of PDS was 0.65% (1.2% in men and 0.0% in women) in the healthy controls, whereas it was 7% (14.3% in men and 3.6% in women) in the symptomatic RKOA (*P* = 0.0083). As the frequency of the signals was near zero in the healthy control, there is no need to establish a normal range and cut-off.

**Table I:** Characteristics of the study population

	Healthy control‡	Symptomatic RKOA§	*P*-value*
*N*	163	44	
Age (years), mean (SD)	65.73 (9.26)	67.23 (9.00)	0.3380
Women, *n* (%)	75 (46.01)	29 (65.91)	0.0192
BMI (kg/m^2^), mean (SD)	25.30 (3.59)	29.73 (6.00)	<0.0001
Effusion right, median (IQR)	4 (2.5–6.7)	8.9 (5.8–12.8)	<0.0001
Synovial hypertrophy right, median (IQR)	0 (0–3.5)	4.45 (1.9–8.4)	<0.0001
Power Doppler Signal right, *n* (%)	1 (0.65)	3 (7.14)	0.0083
Knee pain ever†, *n* (%)	58 (35.58)	44 (100.0)	<0.0001
Knee pain in the past 12 months, *n* (%)	13 (7.98)	41 (93.18)	<0.0001
Global radiographic score (0–60)‖, mean (SD)	2.06 (2.50)	16.47 (7.13)	<0.0001

Note: * *P*-values: *t* test for age, BMI, global radiographic score, Mann–Whitney *U* test for effusion and synovial hypertrophy, and chi-square for categorical unless otherwise specified.

SD – standard deviation; IQR – inter-quarter range; NRS – numerical rating scale 0–10.

† Pain in or around a knee on most days for at least a month.

‡ No current knee pain (knee pain on most days of the past month) and no RKOA (definite JSN (grade 2) plus definite osteophyte (grade 2) in any compartment (tibiofemoral or patellofemoral)).

§ Knee pain on most days of the past month plus RKOA (definite JSN (grade 2) plus definite osteophyte (grade 2) in any compartment (tibiofemoral or patellofemoral)).

‖ Summated score for osteophytes and JSN (NLDLDA scoring system) in tibiofemoral and patellofemoral joints (medial and lateral compartments).

In the healthy controls both effusion and synovial hypertrophy did not associate with age (Appendix 7), but were greater in men than in women (median effusion 4.7 mm in men vs 3.4 mm in women, *P* = 0.0035; median synovial hypertrophy 2.0 mm in men vs 0 mm in women, *P* = 0.0012). The gender difference remained significant after adjustment for height for synovial hypertrophy (*P* = 0.019), but not for effusion (*P* > 0.05). There was no difference between right and left knees in both men and women (all *P* > 0.05).

The distribution of effusion and synovial hypertrophy measurements with a superimposed normal curve in men and women are shown in Appendix 8-1. Because of the high number of zero-values the transformation attempts were unsuccessful (Appendix 8-2). Therefore, original data were used for the analysis.

### “Normal” range

The normal ranges for effusion and synovial hypertrophy in men and women are shown in Table II and Figure 3. The “normal” range of effusion and synovial hypertrophy in a “healthy” sample defined as no KP plus K&L grade 0–1 alternatively are presented in Appendix 9.

**Table II:** “Normal” range of effusion and synovial hypertrophy in mm in people without KP and RKOA

	Men (*n* = 88)				Women (*n* = 75)			
	Min	Max	Median (IQR)	Normal range (0–95th percentile)	Min	Max	Median (IQR)	Normal range (0–95th percentile)
Effusion	0	14.6	4.7 (3.0; 7.2)	0–10.3	0	13.3	3.4 (2.2; 5.5)	0–9.8
Synovial hypertrophy	0	8.2	2.0 (0; 3.8)	0–6.8	0	8.0	0 (0; 2.3)	0–5.4

Note: The 95% CIs for the upper limit of the normal range, i.e., the 95th percentile for effusion were 9.3 mm–14.6 mm in men and 7.4 mm–13.3 mm in women; and those for synovial hypertrophy were 5.8 mm–8.2 mm in men and 3.9 mm–8.0 mm in women.

*P*-values for the difference in effusion and synovial hypertrophy between men and women were 0.0035 and 0.0012, respectively (Mann Whitney *U* Test).

**Fig. 2:**
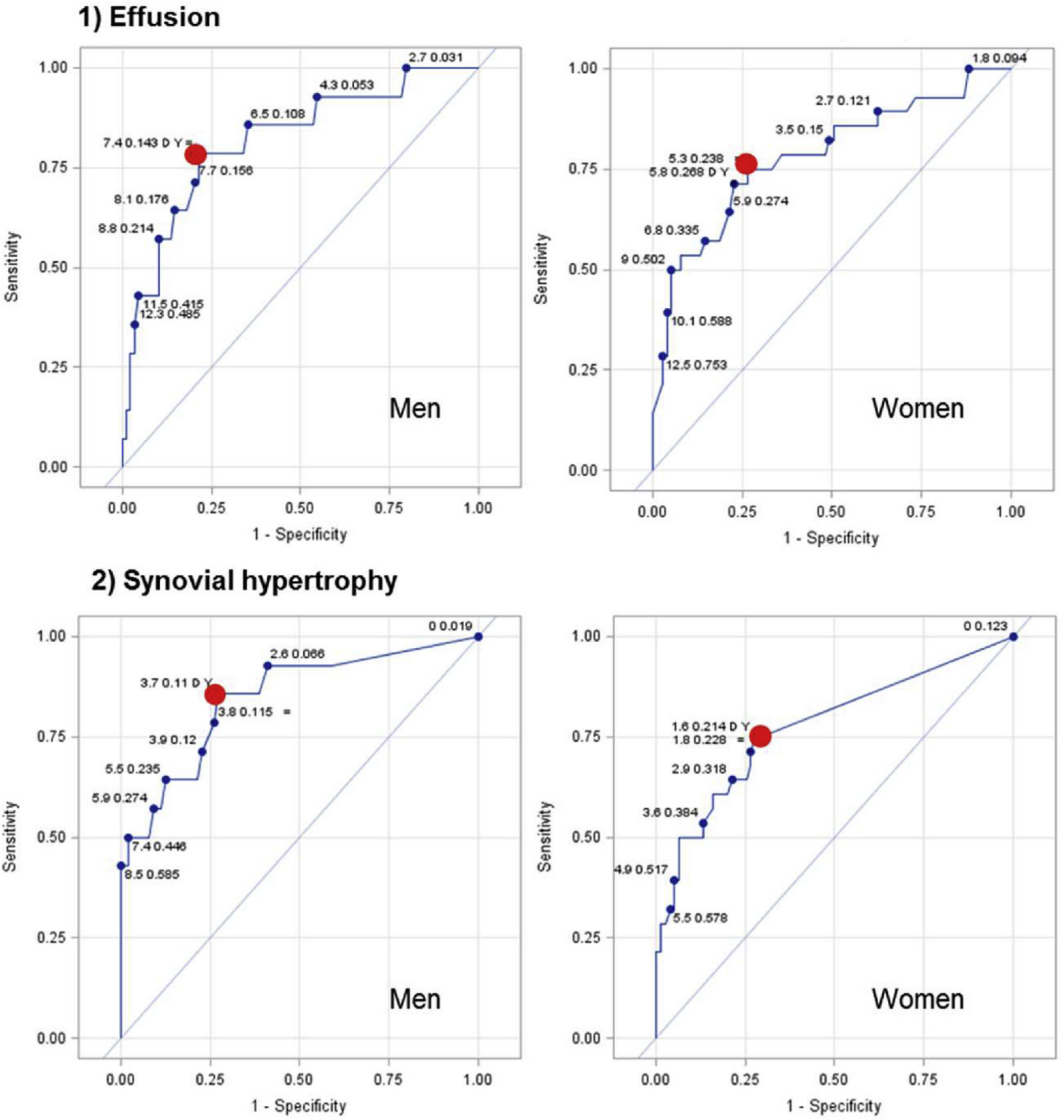
ROC curves for a continuous value of effusion (upper row) and hypertrophy (bottom row) in men (left) and women (right) for discriminating people with symptomatic RKOA from pain-free people without ROA. The red dot represents an optimal cut-off value with the highest Youden Index.

**Fig. 3:**
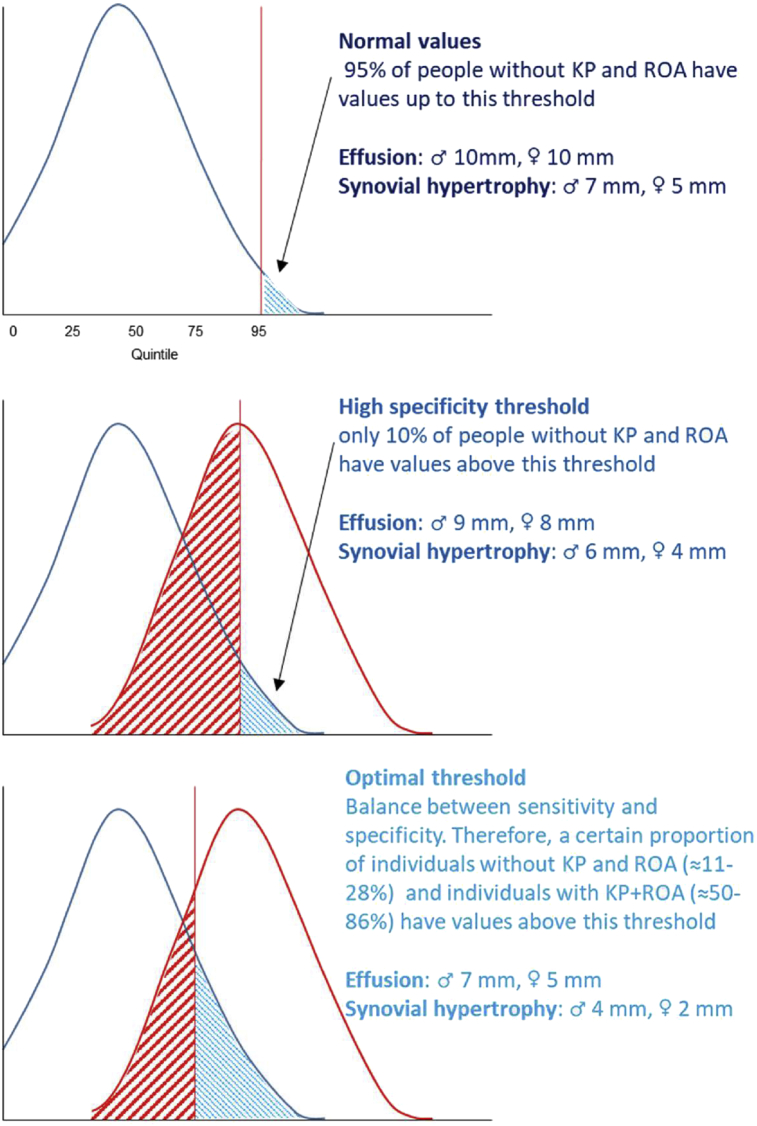
A summary of normal values, high specificity and optimal thresholds for effusion and synovial hypertrophy.

### Different cut-offs: exploring misclassification rate

#### EULAR cut-off

We examined how well the EULAR threshold of 4 mm^18^ separates cases and controls. For effusion the sensitivity of this threshold was 93% and 79% for men and women, respectively but the specificity was only 39% and 61%, respectively. Consequently, 60% of men and 39% of women without the disease were misclassified as having the disease (false-positive), and 7% of men and 21% of women with the disease were misclassified as non-disease (false-negative). For hypertrophy the sensitivity was only 64% and 50% for men and women, respectively, while the specificity was 78% and 89%, respectively. The proportion of men and women with false-positive and false-negative results is shown in Appendix 10.

#### Optimal threshold

Based on the maximum value of Youden Index the optimal threshold for effusion was 7.4 mm in men and 5.3 mm in women, and for synovial hypertrophy it was 3.7 for men and 1.6 for women (Table III and Fig. 3). These new cut-off points were characterised by the maximum sensitivity and maximum specificity of the tested US features (Fig. 2).

**Table III:** Sensitivity, specificity, positive and negative likelihood ratio of synovial effusion and hypertrophy for the diagnosis of knee abnormality (KP plus RKOA) according to the different thresholds

Criterion	Description	Cut-off (mm)	Positive in KP + RKOA∗ group, n/N (%)	Positive in controls, n/N (%)	Sensitivity (95% CI)	Specificity (95% CI)	J	LR+	LR−	FPP	FNP
**Effusion**											
Men	EULAR	4	13/14 (92.86)	54/88 (61.36)	0.93 (0.66; 1.00)	0.39 (0.28; 0.50)	0.31	1.51	0.18	0.81	0.03
	Optimal	7.4	11/14 (78.57)	19/88 (21.54)	0.79 (0.49; 0.95)	0.78 (0.68; 0.86)	0.57	3.64	0.27	0.63	0.04
	High specificity	8.9	7/14 (50)	9/88 (10.23)	0.50 (0.23; 0.77)	0.90 (0.81; 0.95)	0.40	4.89	0.56	0.56	0.08
Women	EULAR	4	22/28 (78.57)	29/75 (38.67)	0.79 (0.59; 0.92)	0.61 (0.49; 0.72)	0.40	2.03	0.35	0.57	0.12
	Optimal	5.3	14/28 (50)	8/75 (10.67)	0.75 (0.55; 0.89)	0.73 (0.62; 0.83)	0.48	2.81	0.34	0.49	0.11
	High specificity	7.8	15/28 (53.57)	7/75 (9.33)	0.54 (0.34; 0.72)	0.91 (0.82; 0.96)	0.44	5.74	0.51	0.32	0.16
**Hypertrophy**											
Men	EULAR	4	9/14 (64.29)	19/88 (21.59)	0.64 (0.35; 0.87)	0.78 (0.68; 0.86)	0.43	2.98	0.46	0.68	0.07
	Optimal	3.7	12/14 (85.71)	24/88 (27.27)	0.86 (0.57; 0.98)	0.73 (0.62; 0.82)	0.58	3.14	0.20	0.67	0.03
	High specificity	5.8	8/14 (57)	9/88 (10.23)	0.57 (0.29; 0.82)	0.90 (0.81; 0.95)	0.47	5.59	0.48	0.53	0.07
Women	EULAR	4	21/28 (75)	20/75 (26.67)	0.50 (0.31; 0.69)	0.89 (0.80; 0.95)	0.39	4.69	0.56	0.36	0.17
	Optimal	1.6	21/28 (75)	21/75 (28)	0.75 (0.55; 0.89)	0.72 (0.60; 0.82)	0.47	2.68	0.35	0.50	0.11
	High specificity	4.2	14/28 (50)	7/75 (9.33)	0.50 (0.31; 0.69)	0.91 (0.82; 0.96)	0.41	5.36	0.55	0.33	0.17

Abbreviations: J – Youden Index; “LR−” – likelihood ratio of a negative test result; FPP – false positive probability; FNP – false negative probability.

∗ According to NDLA for current, optimal and high sensitivity thresholds (NLDLDA).

#### Threshold with high specificity

For effusion the threshold corresponding with specificity of 90% was 8.9 mm in men and 7.8 mm in women (Table III and Fig. 3). For synovial hypertrophy the threshold corresponding with high specificity was 5.8 in men and 4.2 in women. The LR+ for these cut-offs was close to 5 (higher than LR+ for other cut-offs).

All three cut-off values with corresponding sensitivity, specificity, and other measures of diagnostic accuracy are presented in Table III and Figure 3.

## Discussion

To our knowledge, this is the first population-based study in an age-range suitable for knee OA to investigate reference values and the cut-off of USSCs for identification of symptomatic RKOA. The main findings are: [1] USSCs are different between men and women therefore gender-specific reference limits should be established; [2] the “normal” range for effusion is between 0–10.3 mm for men and 0–9.8 mm for women and the “normal” range for synovial hypertrophy is between 0–6.8 mm for men and 0–5.4 mm for women; [3] the optimal cut-off that may be used to screen people with abnormally increased synovial changes in symptomatic RKOA is 7 mm for men and 5 mm for women for effusion, and 4 mm for men and 2 mm for women for hypertrophy; [4] the more stringent cut-off with high specificity that may be more appropriate for defining “active” cases for RCTs and for identifying a more inflammatory (endo)phenotype of symptomatic RKOA is 9 mm for men and 8 mm for women for effusion, and 6 mm men and 4 mm for women for hypertrophy.

No previous studies have reported reference values for US in the general population aged over 40 years old. Recently a large study of D'Agostino *et al.*^37^ reported a high prevalence of USSCs in a population-based cohort aged >60 years old (effusion present in 69.7% and synovial hypertrophy in 53.1%). However, no data on distribution (mean values, min–max range) were reported. Nevertheless, the high prevalence of US features in this cohort is in line with our results. The “normal” range was established for men and women separately as we found significantly higher values of synovial hypertrophy in men compared to women. Our results are in line with the study of D'Agostino *et al.*^18^ that reported that women had fewer joint effusions than men (OR 0.62). The subgroup-based “normal” range provides more sensitive and specific results and improves clinical application^38^. Moreover, the larger values in men align with thicker cartilage in men and the development of different ranges in men and women for radiographic assessment using the (LDLDA)^27^, ^28^.

It is important to recognise the difference between the reference intervals and cut-offs. The reference interval is the range of values that would reflect a biological variability of a diagnostic marker in a healthy population. Typically, reference intervals are referred to as “normal” values and therefore any test result would be interpreted relative to its upper (or lower) limit. However, for many diagnostic tests “normal” values have been defined on the basis of analysis of clinical outcomes^39^. Cut-offs (“decision limits”) depend on the type of pathological condition being considered and the type of decision to be made^40^. For example, the 97.5 percentile for cholesterol concentration in the general population lies between 280 and 300 mg dL^−1^ (7.25–7.77 mmol L^−1^), while the cut-off associated with moderate and high risks for the development of cardiovascular disease are 200 mg dL^−1^ (5.18 mmol L^−1^), and 240 mg dL^−1^ (6.22 mmol L^−1^), respectively (National Cholesterol Education Program (NCEP) Expert Panel^41^). Therefore, in this study in addition to the reference intervals for effusion and hypertrophy in pain-free participants without RKOA, we calculated cut-offs corresponding with the presence of symptomatic RKOA. Because of the large overlap between people with and without symptomatic RKOA, we applied two different methods to establish a cut-off. Firstly, we calculated an optimal cut-off using the Youden Index. This method has been widely used to identify an optimal cut-off with maximum sensitivity and specificity^42^, ^43^. Secondly, we calculated a threshold corresponding with pre-defined specificity at 90% to identify a subgroup of people with symptomatic RKOA who are different from the healthy population. These cut-offs corresponded with the highest likelihood ratio of a positive test result (“LR+” ≈ 5). This subgroup is more likely to represent an “inflammatory” phenotype.

In our study the prevalence of PDS was very low in the healthy control group and higher in people with symptomatic RKOA. Two studies previously reported prevalence of PDS in the general population^37^, ^44^. In the study by Hall *et al.*^44^ the prevalence of PDS in pain-free people without RKOA (*n* = 90) was 2.2% and in people with symptomatic RKOA 16.2%, which is in line with our findings. In the study by D'Agostino *et al.*^37^ the prevalence of PDS was 31.8% in the general population. However, this cohort (*n* = 433) was older (range 60–98) and the prevalence of KP was 31.6%.

There are several limitations to this study. Firstly, KPIC is a questionnaire-based cohort study for KP, therefore, participants with KP may be more likely to respond to the study (response bias). Secondly, sampling bias cannot be discounted. Although we randomly selected participants for this study from the KPIC cohort, people with KP are generally more willing to participate in a clinical assessment (prevalence of KP was 21% in non-responders and 30% in responders, *P* = 0.036, Appendix 3). Sampling bias also could account for the unrepresentativeness of the younger age group (less than 55) as the working age population is less likely to respond to the invitation. Thirdly, we used “current KP” definition to divide our sample into those with and without KP in order to determine the decision threshold for both US values. This definition is one of the clinical criteria for knee OA according to the American College of Rheumatology (ACR)^45^. A study by O'Reilly *et al.*^24^ which compared different questions on KP showed that this definition is the most specific (72.7%) but least sensitive (45.4%) predictor of disability because of KP. However, applying a different KP definition would lead to a different decision threshold. Fourthly, pain, USSCs and RKOA were measured at one time point and longer follow-up with repeat measures might have allowed better discrimination and predictive value. Fifthly, diagnostic accuracy is affected by the characteristics of the population in which the test accuracy is evaluated such as the disease prevalence or the spectrum of the disease. Further validation is needed when attempting to use the reference intervals and decision limit produced from this study. Furthermore, we used KP plus RKOA as our reference standard to define cases and control. Whether this is an adequate “gold standard” for the USSCs examined requires further investigation. It is suggested that the three USSCs are all features of “synovitis”. However, KP is not only caused by “synovitis” and RKOA is often asymptomatic and non-inflammatory. The large overlapping between cases and controls for the USSCs may suggest that our reference standard needs to be improved. Further study using MRI synovitis as a reference standard may be useful.

In summary, this study suggests that effusion and synovial hypertrophy but not PDS are common in the general population including people without KP and RKOA. Different thresholds for both effusion and synovial hypertrophy should be applied for men and women. These data are useful for the classification of synovial abnormalities in people with symptomatic RKOA, and the development/revision of evidence based guidelines such as the EULAR recommendations for the US abnormalities in knee OA.

## Registration

This study was approved by the Nottingham Research Ethics Committee 1 (Ref 15/EM/0529) and by the Nottingham University Hospitals Research and Innovation Department (Ref 15RH015).

## Authors' contributions

AS, WZ and MD made substantial contributions to the conception and design of the study. All authors contributed to the acquisition of questionnaire data. US examination was performed by AS and MH; scoring by the LDLDA scoring system was performed by AS and GSF. AS, MD and WZ conducted the data analysis and interpretation. AS wrote the first draft. WZ has full access to the data and takes responsibility for the content and guarantees the integrity and accuracy of the work undertaken. All authors have read, provided critical feedback on intellectual content and approved the final manuscript.

## Conflict of interest disclosures

All authors have completed and submitted the ICMJE Form for Disclosure of Potential Conflicts of Interest.

## Funding source

This work was financially supported by the University of Nottingham (ref RGS 15088) and by Arthritis Research UK (Pain Centre Initiative grant number: 20777).

## Role of the funder/sponsor

The sponsor did not participate in the design and conduct of the study; collection, management, analysis, and interpretation of the data; or preparation, review, or approval of the manuscript and the decision to submit the manuscript for publication.

## Disclaimer

The opinions, results and conclusions reported in this article are those of the authors and are independent from the funding sources.
