# Supplementary material for: Thresholds of ultrasound synovial abnormalities for knee osteoarthritis – a cross sectional study in the general population
Source: Osteoarthritis Cartilage. 2019 Mar;27(3):435–43. doi: 10.1016/j.joca.2018.09.018 (PMC6414397; doi:10.1016/j.joca.2018.09.018)
Supplement: Multimedia component 1 [file mmc1.docx]

Table of Contents

[Appendix 1. Characteristics of practices selected for recruitment. 2](#_Toc519515717)

[Appendix 2. Characteristics of the random sample from the general population 3](#_Toc519515718)

[Appendix 3. Clinical characteristics of the study population 4](#_Toc519515719)

[Appendix 4. Grey-scale US image of an effusion and synovial hypertrophy in the supra-patellar pouch, and Power Doppler signal in the lateral tibio-femoral space of the knee. 5](#_Toc519515720)

[Appendix 5. Establishing “normal” range and an optimal threshold (detailed statistical methods) 6](#_Toc519515721)

[Appendix 6. Missing data 12](#_Toc519515722)

[Appendix 7. The mean US measures of effusion and hypertrophy in mm (95% CI) in men and women - comparison across different age groups 13](#_Toc519515723)

[Appendix 8. Raw US effusion/hypertrophy distribution and transformation attempts 14](#_Toc519515724)

[Appendix 9. Alternative ROA definition (current KP + K&L≥2) 16](#_Toc519515725)

[Appendix 10. The probability density functions of a continuous value of effusion (upper row) and hypertrophy (bottom row) for people with KP and ROA (red dashed line) and pain-free people without ROA (blue solid line) persons. 17](#_Toc519515726)

# Appendix 1. Characteristics of practices selected for recruitment.

|  | Selected practices ** | Other practices** | All practices* |
| --- | --- | --- | --- |
| Total number of responders | 1662 | 3017 | 4679 |
| Agreed to receive information | 1284 | 2331 | 3615 |
| Mean age (SD***) | 63.49 (10.07) | 61.03 (9.91) | 61.93 (10.04) |
| Proportion of females, (%) | 765 (59.61) | 1364 (58.55) | 2130 (58.92) |
| Mean BMI (SD***) | 26.61 (5.00) | 27.77 (5.49) | 27.35 (5.35) |
| Prevalence of KP, (%) | 735 (57.25) | 1435 (61.58) | 2169 (60.00) |

**Notes:**

* - This is the source population.

** - **Selected practices**: Family Medical Centre; Keyworth Medical Practice; Rivergreen; Medical Centre; Hucknall Road Medical Centre; Churchfields Medical Practice.

**Other practices**: Heartwood Medical Practice, Collingham Medical Centre, Church Walk Surgery, The Park Surgery, Gladstone House surgery, Hill View Surgery’ Bilsthorpe Surgery.

*** - SD – standard deviation.

# Appendix 2. Characteristics of the random sample from the general population

|  | **Total**  **(N=500)** | **Responders**  **(N=360)** | **P-value*** |
| --- | --- | --- | --- |
| **Age (years), mean (SD)** | 63.77 ( 9.83) | 64.70 (9.56) | 0.0006 |
| **Women, n (%)** | 250 (50.00) | 182 (50.42) | 0.8745 |
| **BMI, mean (SD)** | 26.68 ( 4.99) | 26.78 (4.97) | 0.4757 |
| **Knee pain, (%)** | 244 (48.80) | 187 (51.80) | 0.2871 |
| **Current knee pain, (%)** | 138 (27.60) | 109 (30.19) | 0.2702 |
| **Current knee pain severity (NRS 0-10), mean (SD)** | 1.91 ( 2.88) | 1.99 (2.86) | 0.2914 |
| **Worsening of pain at one year**** | 49 ( 9.80) | 35 (9.70) | 0.9466 |

Note: * p-value for the difference between responders and the whole source population;

** - according to the Patient Global Impression of Change.

SD - standard deviation; BMI - body mass index; NRS – numerical rating scale (range 0-10).

# Appendix 3. Clinical characteristics of the study population

|  | **Men** | **Women** | **p-value*** |
| --- | --- | --- | --- |
| **N** | 152 | 147 |  |
| **Age (years), mean (SD)** | 66.64 (9.21) | 65.29 (9.24) | 0.2060 |
| **BMI (kg/m^2^), mean (SD)** | 26.55 (4.47) | 27.03 (5.45) | 0.4025 |
| **Knee pain ever^1^, n (%)** | 89/152 (58.55) | 87/147 (59.18) | 0.9117 |
| **Knee pain in the past 12 months, n (%)** | 56/152 (36.84) | 60/147 (40.82) | 0.4808 |
| **Current knee pain (KP)^2^, n (%)** | 43/152 (28.29) | 49/147 (33.33) | 0.3448 |
| **Current knee pain severity (NRS 0-10), mean (SD)** | 1.64 (2.56) | 2.13 (2.93) | 0.1237 |
| **RKOA^3^, n (%)** | 41/150 (27.33) | 53/143 (37.06) | 0.0745 |
| **KP^2^+RKOA, n (%)** | 14/151 (9) | 28/147 (18) | 0.0248 |
| **Global radiographic score (0‑60)^4^, mean (SD)** | 5.32 (7.27) | 6.10 (7.30) | 0.3572 |

Note: * P-values: t test for continuous and chi-square for categorical unless otherwise specified.

SD - standard deviation; NRS – numerical rating scale 0-10; BMI - body mass index.

^1^ Pain in or around a knee on most days for at least a month.

^2^ Knee pain on most days of the past month.

^3^ RKOA: Radiographic knee OA defined as definite JSN (grade 2) plus definite osteophyte (grade 2) in any compartment (tibiofemoral or patellofemoral).

^4^ Summated score for osteophytes and joint space narrowing (NLDLDA scoring system) in tibiofemoral and patellofemoral joints (medial and lateral compartments).

# Appendix 4. Grey-scale US image of an effusion and synovial hypertrophy in the supra-patellar pouch, and Power Doppler signal in the lateral tibio-femoral space of the knee.


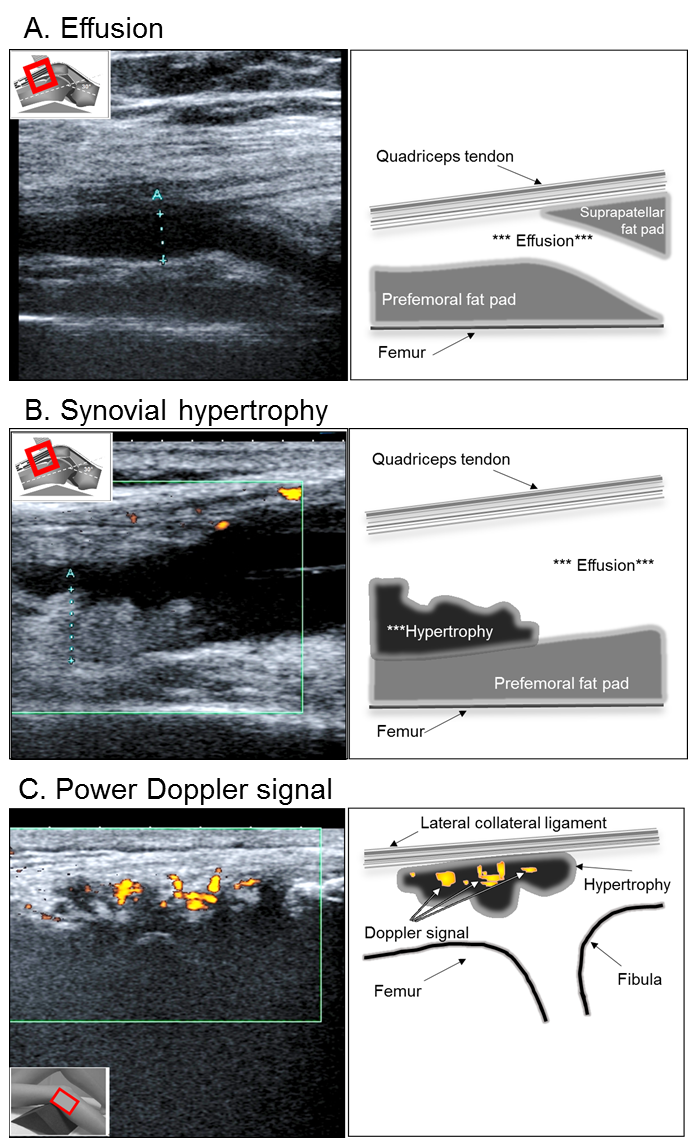


***Note: On the left the real US images with detected synovial pathology (taken from study participant); with images of the knee area and position (adapted from (Bianchi and Martinoli 2007)*, permission granted); on the right are schematic drawings synovial pathology in relation to other joint structures.**

Reference: Bianchi, S. and C. Martinoli (2007). Ultrasound of the musculosceletal system. New York, Springer: 637-745.

# Appendix 5. Establishing “normal” range and an optimal threshold **(detailed statistical methods)**

The results of a diagnostic test can provide an answer to two important questions such as [1] whether this result lies within a range of values in a 'healthy' population *(****“normal” range***) and [2] whether it corresponds with a specific level of risk or probability for the presence of a certain disease (***decision limits***) (Ceriotti and Henny 2008). While the “normal” range is simply a statistical definition of the biological variability of the population, for some diagnostic tests it is more important to refer to the decision limits defined on the basis of analysis of clinical outcomes between “normal” range and disease/”abnormal” range (Murphy and Abbey 1967, Ceriotti and Henny 2008).

**“Normal” range**

The reference interval or “normal” range is an interval between, and including, two reference limits corresponding to 95% of the population of healthy subjects (Ceriotti and Henny 2008). In this study the upper reference limit (95% quintile) for effusion and synovial hypertrophy was calculated in the healthy sample (no current KP and no ROA). Quintiles were calculated using the distribution-free method (“proc UNIVARIATE CIPCTLDF”, SAS) with corresponding 95% CI (Hahn and Meeker 2011). A schematic representation of the reference interval with the upper reference limit and corresponding 95% CI is shown in Figure 5-1.

Figure 5-1. Schematic representation of the reference interval and upper reference limit with its confidence intervals


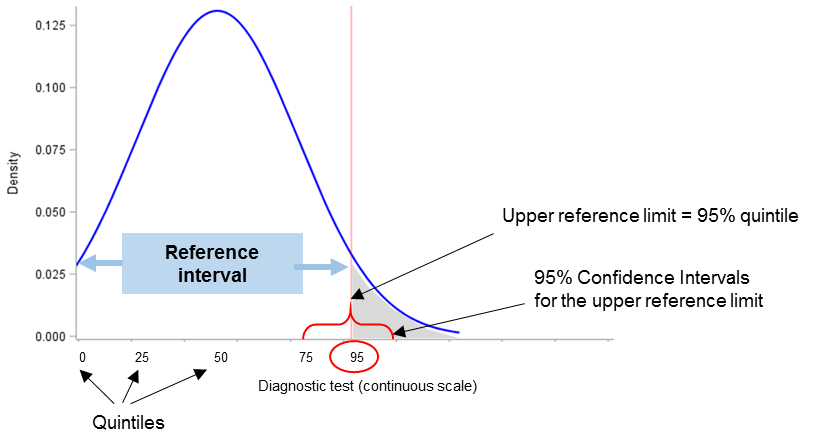


**Optimal threshold**

The discrimination ability (i.e. ability to separate cases and controls) of each synovial change was determined in a case control study, where people with KP and ROA were classified as cases and those without were classified as controls. Because both effusion and synovial hypertrophy were different between men and women, the diagnostic accuracy was examined in men and women separately.

The discrimination ability was examined using the following measures of diagnostic accuracy:

- *Sensitivity* is the proportion of participants with outcome correctly classified as ′diseased′.
- *Specificity* is the proportion of participants without outcome correctly classified as ′non-diseased’.
- *False positive probability (FPP)* is the proportion of positive test results in true negative cases.
- *False negative probability* *(FNP)* is the proportion of negative test results in true positive cases.
- *The likelihood ratio of a positive test result (LR+)* is sensitivity divided by 1-specificity. It describes how the probability of disease shifts when the finding is present.
- *The likelihood ratio of a negative test result (LR-)* describes how the probability of disease shifts when it is absent (1-sensitivity divided by specificity). The magnitude of the LR (range from 0 to infinity) suggests how strongly a given test result will raise or lower the likelihood of disease (McGee 2002).
- *A ROC curve* is a plot of sensitivity versus 1-Specifity which indicates how effectively the test identifies the diseased and non-diseased people. The general structure of a ROC curve is shown in Figure 5-2. There is a diagonal line joining (0, 0) and (1, 1) which represents a random chance to distinguish people with versus those without a disease. If the performance of a diagnostic test is no better than chance level the ROC curve lies on the diagonal line. If a diagnostic test perfectly distinguishes between the diseased and non-diseased people (100% sensitivity and 100% specificity) the ROC curve reaches the upper left corner. Each data point on the graph represents a different cut-off point with corresponding Sensitivity and Specificity.
- *Area Under the Curve (AUC)* is a quantitative summary measure of the ROC curve. Its values range from 0 to 1 where a perfect diagnostic test will have an AUC value of 1, whereas a worthless diagnostic test will have an AUC <=0.5. “ROCPLOT” macros was used to plot ROC curves and calculate associated statistics (6)(<http://support.sas.com/kb/25/018.html>).


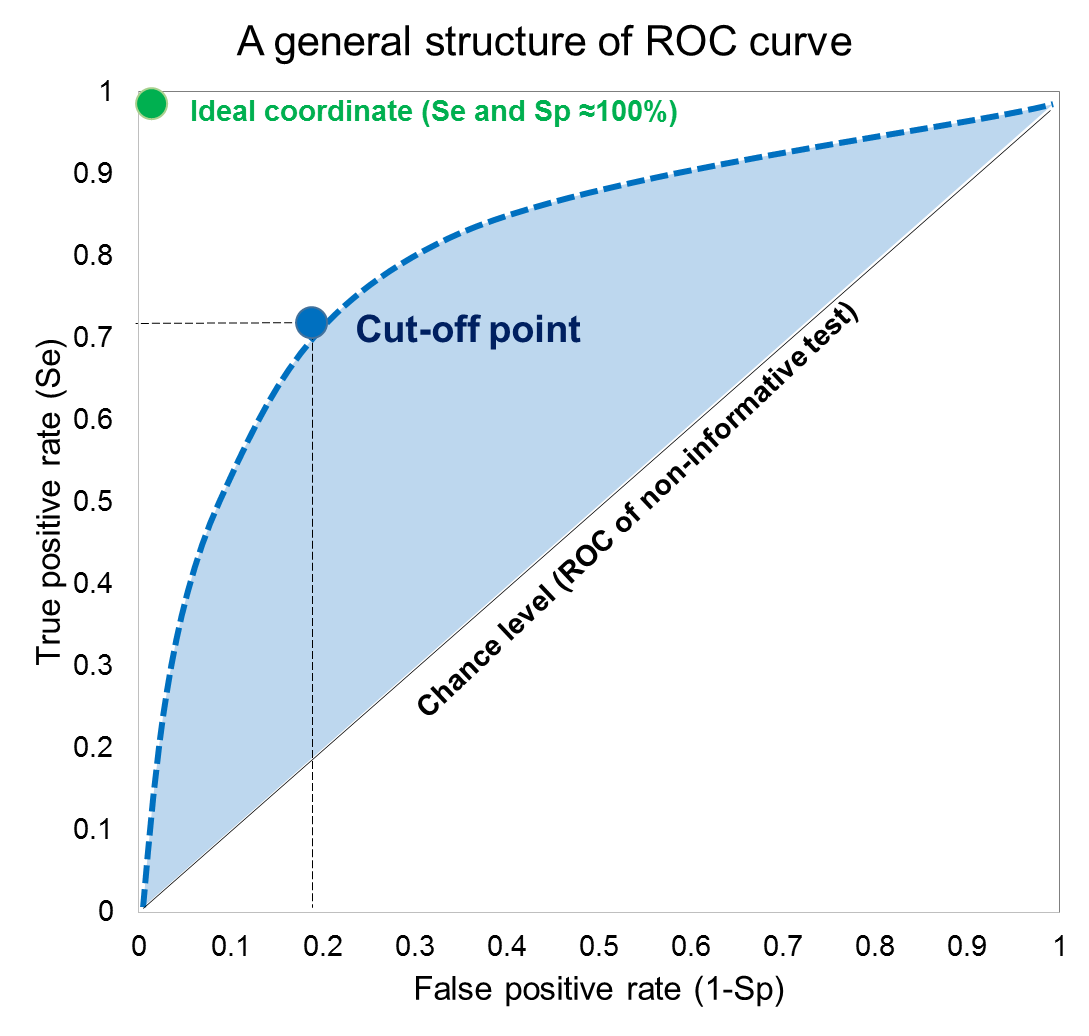


Figure 5-2. A general structure of the ROC curve

The values predicted from the model are presented as the blue dashed curve. The blue shaded area is Area under the Curve. The blue circle indicates the cut-off point with corresponding Sensitivity and Specificity (black dash lines). The green circle is the ideal point of maximum Sensitivity and Specificity. The 45° diagonal line shows the ROC of an uninformative test.

An ideal diagnostic test that has perfect sensitivity and perfect specificity can determine disease status with certainty (i.e., no misclassification) (Figure 5-3, left image). However, in the real world almost all tests to some extent miss disease or indicate disease in normal people (Figure 5-3, image B, C, D). The relative importance of a false negative versus a false positive diagnosis varies according to the diagnostic tests and disease of interest (Mallett, Halligan et al. 2012). Therefore, for many diagnostic tests, there are multiple potential thresholds. For example, if the diagnostic test is used for screening a life threatening disease, a more sensitive but less specific cut-off is preferable because missing a case is regarded as much more important than making a false positive diagnosis in a healthy person (Mallett, Halligan et al. 2012). However, a more specific but less sensitive threshold is preferable when a diagnostic test is used to select people who represent a particular subgroup (“phenotype”) which is different from the general population.


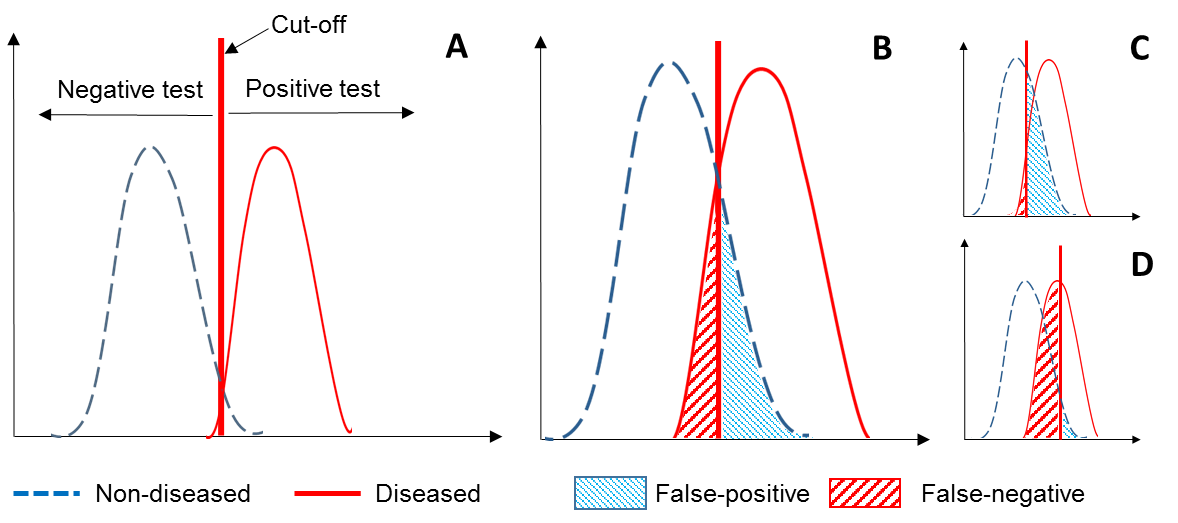


***Figure 5-3. Different decision thresholds.***

*A cut-off value is represented by the vertical red line. All test values equal or greater than this value are considered positive, otherwise they are considered negative.*

*A. A diagnostic test with perfect sensitivity and perfect specificity.*

*B, C, D. The distribution curves overlap meaning that the diagnostic test cannot fully separate diseased and non-diseased people. If the test indicates disease in normal people, these people are false-positives (over-diagnosed). Those people with disease classified by the test as negative are false-negatives (missed cases).The cut-off “B” is corresponding with maximum sensitivity and specificity. As the cut-off value decreases (C), the test Sensitivity increases and the test Specificity decreases. Increasing the cut-off value (D) will give you a more specific but less sensitive test.*

Three **cut-offs** were identified and examined in this study:

- Youden index: A threshold with the maximum sensitivity and specificity (*J = Maximum (Sensitivity+ Specificity -1)* (Habibzadeh, Habibzadeh et al. 2016). The results range between 0 to 1, where J=1 indicates that there is no false-negative or false-positive values, and J=0 indicates that the diagnostic test cannot differentiate between diseased and non-diseased subjects.
- A threshold with a relatively high specificity of 90% to ensure the minimum misdiagnosis.
- A threshold of 4 mm recommended by EULAR (D'Agostino, Conaghan et al. 2005).

References:

"Plot ROC curve with cutpoint labeling and optimal cutpoint analysis." Retrieved 7 June 2017, from http://support.sas.com/kb/25/018.html.

Ceriotti, F. and J. Henny (2008). "“Are my Laboratory Results Normal?” Considerations to be Made Concerning Reference Intervals and Decision Limits." EJIFCC 19(2): 106-114.

D'Agostino, M. A., P. Conaghan, M. Le Bars, G. Baron, W. Grassi, E. Martin-Mola, R. Wakefield, J. L. Brasseur, A. So, M. Backhaus, M. Malaise, G. Burmester, N. Schmidely, P. Ravaud, M. Dougados and P. Emery (2005). "EULAR report on the use of ultrasonography in painful knee osteoarthritis. Part 1: prevalence of inflammation in osteoarthritis." Annals of the Rheumatic Diseases 64(12): 1703-1709.

Habibzadeh, F., P. Habibzadeh and M. Yadollahie (2016). "On determining the most appropriate test cut-off value: the case of tests with continuous results." Biochemia Medica 26(3): 297-307.

Hahn, G. J. and W. Q. Meeker (2011). Statistical intervals: a guide for practitioners, John Wiley & Sons.

Mallett, S., S. Halligan, M. Thompson, G. S. Collins and D. G. Altman (2012). "Interpreting diagnostic accuracy studies for patient care." BMJ 345: e3999.

McGee, S. (2002). "Simplifying Likelihood Ratios." Journal of General Internal Medicine 17(8): 647-650.

Murphy, E. A. and H. Abbey (1967). "The normal range--a common misuse." J Chronic Dis 20(2): 79-88.

# Appendix 6. Missing data

| **Variable name** | **N of missing** |
| --- | --- |
| Age (years), mean (SD) | 0 |
| Gender | 0 |
| BMI (kg/m^2^) | 4 |
| Knee pain | 0 |
| Current knee pain | 0 |
| ROA | 6 |
| Global radiographic score (0‑60) | 6 |
| Effusion | 2 |
| Hypertrophy | 2 |
| Doppler signal | 2 |

# Appendix 7. The mean US measures of effusion and hypertrophy in mm (95%
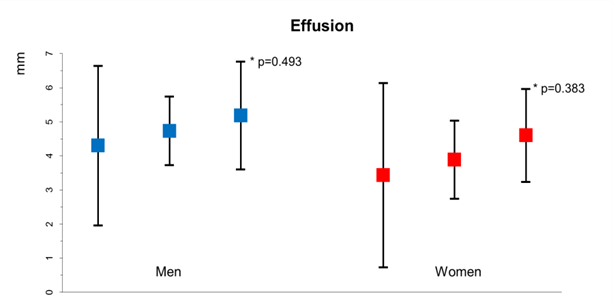

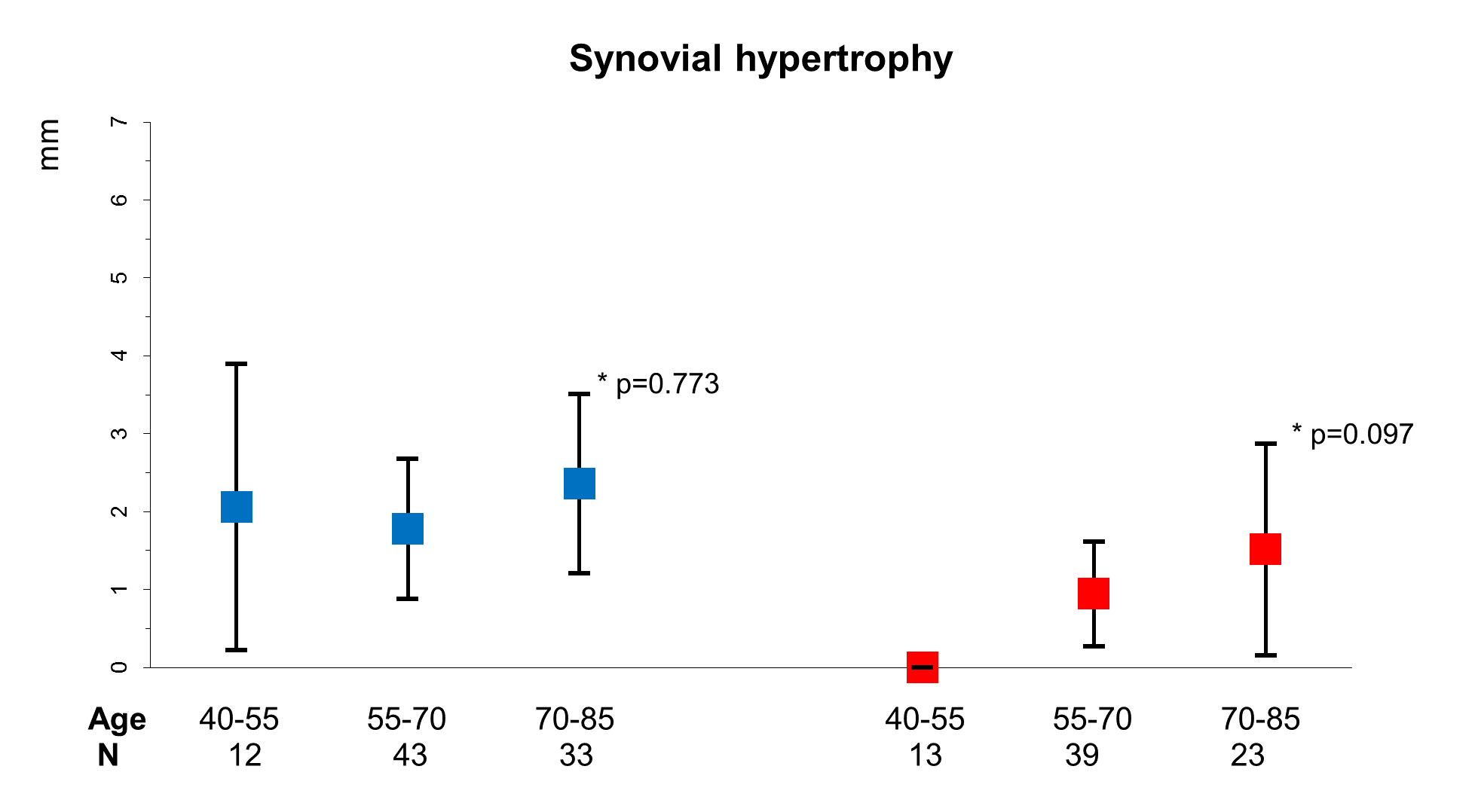
CI) in men and women - comparison across different age groups

Note:* P-value for trend.

# Appendix 8. Raw US effusion/hypertrophy distribution and transformation attempts

1. Raw US effusion/hypertrophy distribution by gender

**Effusion**


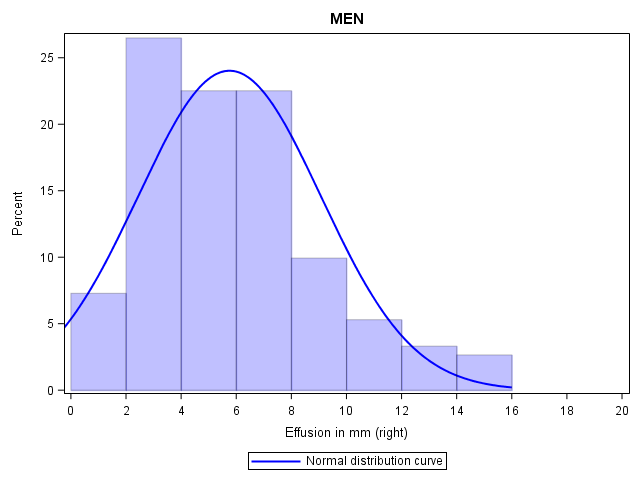


Men


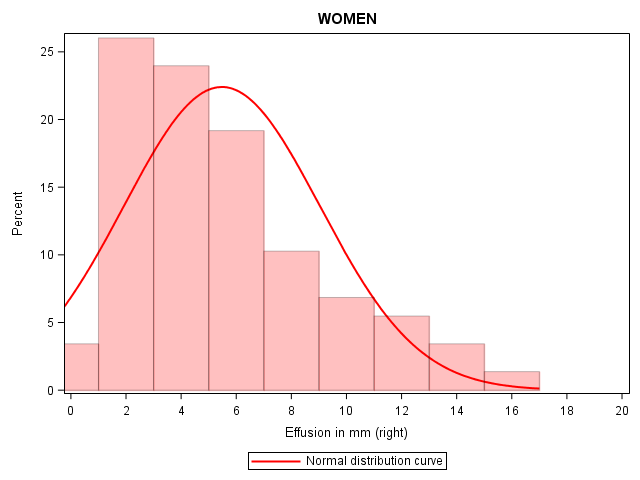


Women


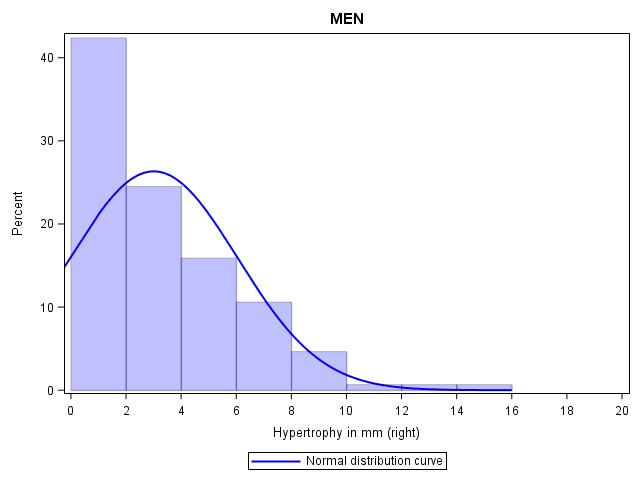


Men


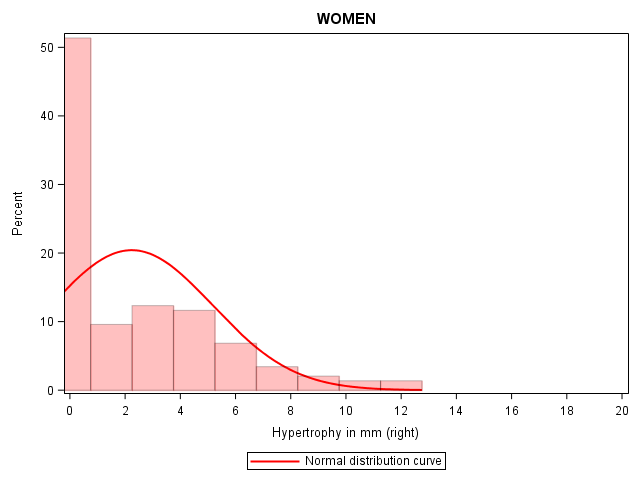


Women

**Synovial hypertrophy**

1.
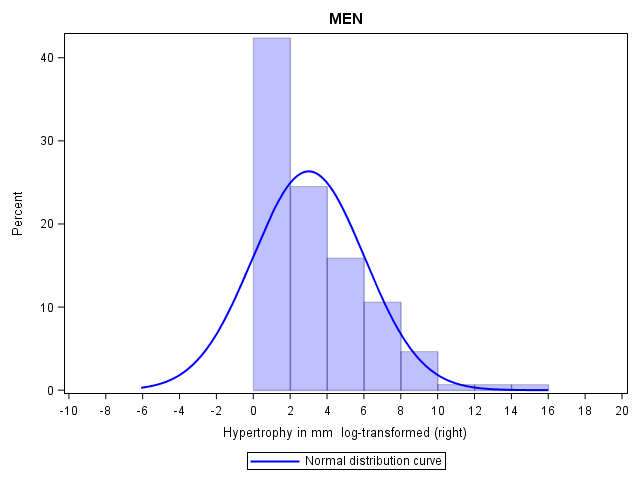

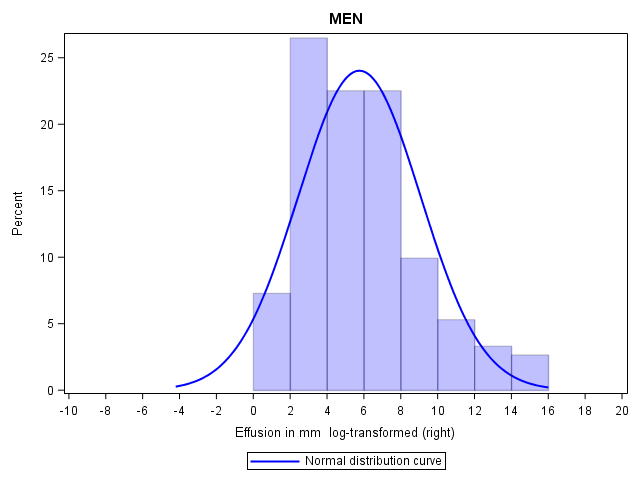
Log-transformation


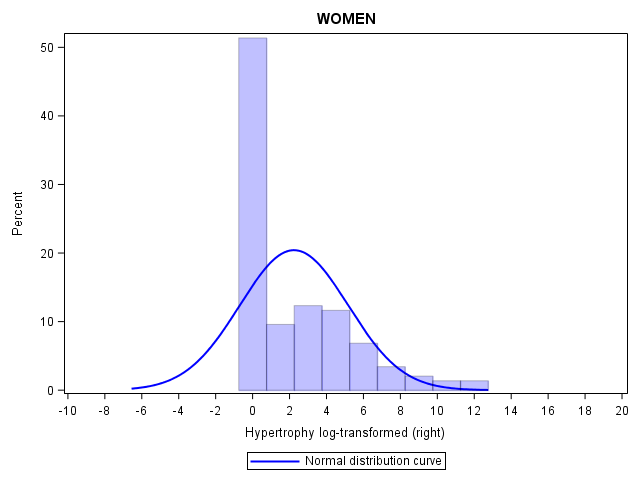

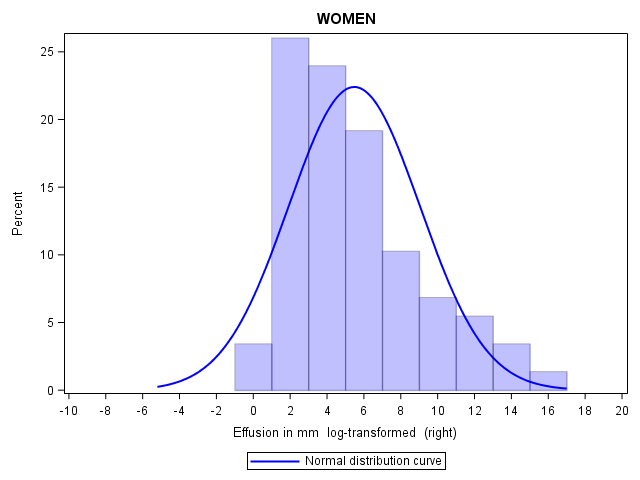


Other transformations

Men


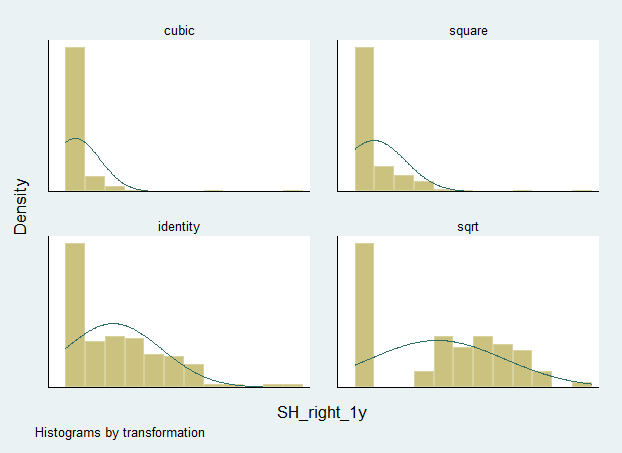

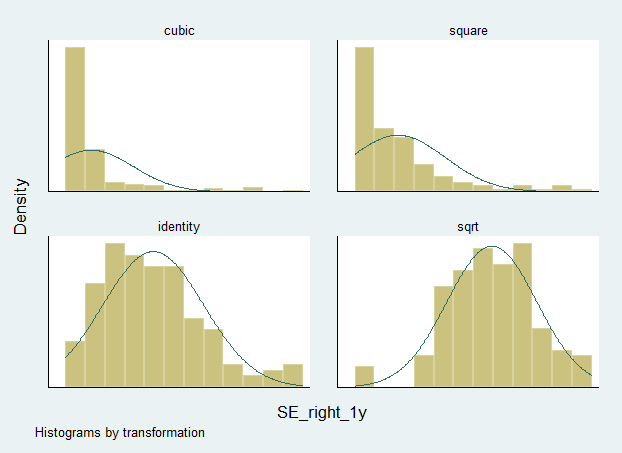


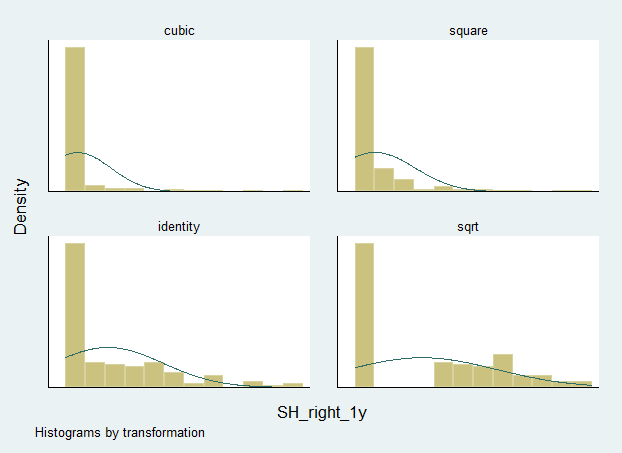

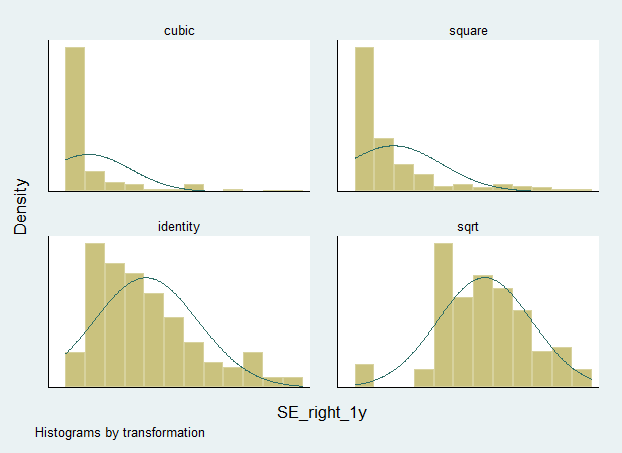
Women

# Appendix 9. Alternative ROA definition (current KP + K&L≥2)

|  | **Men (n=84)** | | | **Women (n=64)** | | |
| --- | --- | --- | --- | --- | --- | --- |
|  | **Range (min‑max)** | **Mean (SD)** | **Normal range** | **Range (min‑max)** | **Mean (SD)** | **Normal range** |
| *Effusion* | 0-14.6 | 5.3 (3.1) | 0 - 10.3 | 0-9.8 | 3.7 (2.3) | 0 - 7.8 |
| *Synovial hypertrophy* | 0-8.2 | 2.3 (2.4) | 0 - 6.9 | 0-8.0 | 0.9 (1.8) | 0 - 4.8 |

Note: Confidence limits for the 95^th^ percentile are for effusion from 9.4 mm to 14.6 mm in men and from 7.4 mm to 9.8 mm in women; and for synovial hypertrophy from 5.8 mm to 8.2 mm in men and from 4.1 mm to 8.0 mm in women

#
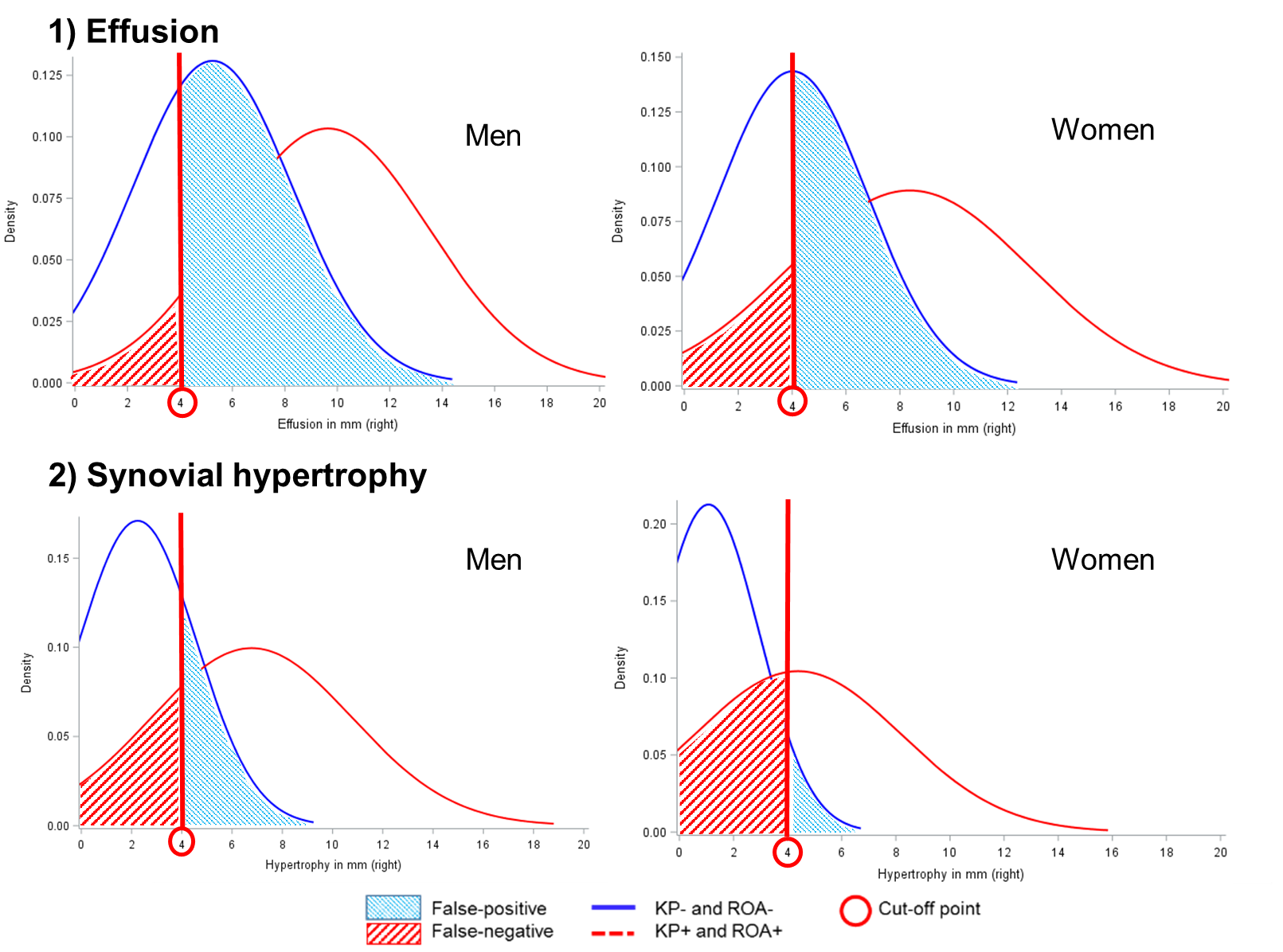
A**ppendix 10. The probability density functions of a continuous value of effusion (upper row) and hypertrophy (bottom row) for people with KP and ROA (red dashed line) and pain-free people without ROA (blue solid line) persons.**

Note: The cut-off value is represented by the vertical red line. All test values equal or greater than this value are considered positive, else they are considered negative. The area under the density functions shaded with red diagonal lines to the left of the cut-off value is False-negative rate, and the area shaded with blue color to the right of the cut-off value is False-positive rate.
